# Device Data Ingestion for Industrial Big Data Platforms with a Case Study †

**DOI:** 10.3390/s16030279

**Published:** 2016-02-26

**Authors:** Cun Ji, Qingshi Shao, Jiao Sun, Shijun Liu, Li Pan, Lei Wu, Chenglei Yang

**Affiliations:** 1 School of Computer Science & Technology, Shandong University, Jinan 250101, China; jicun@sdu.edu.cn (C.J.); sdusqs@163.com (Q.S.); jiaosun_baby@163.com (J.S.); panli@sdu.edu.cn (L.P.); i_lily@sdu.edu.cn (L.W.); 2 Engineering Research Center of Digital Media Technology, Shandong University, Jinan 250101, China; 3 Beijing Key Laboratory on Integration and Analysis of Large-scale Stream Data, North China University of Technology, Beijing 100144, China

**Keywords:** big data, internet of things, industrial internet of things, device data ingestion

## Abstract

Despite having played a significant role in the Industry 4.0 era, the Internet of Things is currently faced with the challenge of how to ingest large-scale heterogeneous and multi-type device data. In response to this problem we present a heterogeneous device data ingestion model for an industrial big data platform. The model includes device templates and four strategies for data synchronization, data slicing, data splitting and data indexing, respectively. We can ingest device data from multiple sources with this heterogeneous device data ingestion model, which has been verified on our industrial big data platform. In addition, we present a case study on device data-based scenario analysis of industrial big data.

## 1. Introduction

**Figure 1:**
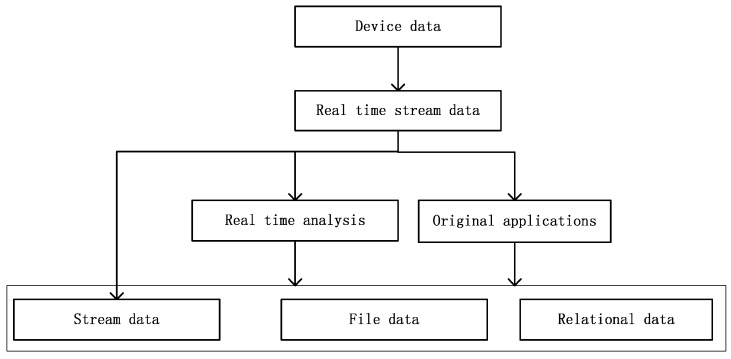
Multi-source data used as device data.

The Internet of Things (IoT) has been defined as communication between and integration of smart objects (things) [1]. Nowadays, the rapid development of IoT has aroused increasing interest in a variety of fields [2]. In the industrial field especially, more and more sensors are being incorporated into smart products, manufacturing equipment, and production monitoring. Smart manufacturing, which has become a vital component of manufacturing in the Industry 4.0 era, is currently facing challenges related mainly to the following three issues:

1. Heterogeneous data. There are different types of smart devices in an enterprise. Each device has single-parameter or multiple-parameter sensors, resulting in the creation of heterogeneous data. The first big challenge is how to ingest heterogeneous data.
2. Multi-source data. Apart from real-time streaming data, there are also legacy applications managing existing devices in enterprises. It is unrealistic to expect enterprises to stop using such applications completely. Moreover, we can analyze real-time streaming data in real-time. As shown in Figure 1, after real-time analysis and original application, device data may be streaming data, file data or data in relational databases. How to ingest multi-source data is the second big challenge.
3. Big data. With the development of smart manufacturing technology, it can be foreseen that the IoT will increase the scale of data to an unprecedented level [3]. Recently, we have witnessed explosive growth in the variety, velocity, and volume of data [4]. The era of big data has arrived [5,6,7,8]. More effective approaches for resolving record storage and queries in a big data environment are required.

To solve the above issues, a heterogeneous device data ingestion model is urgently needed. Unfortunately, existing models do not properly address these issues. Therefore, we propose a heterogeneous device data ingestion model for our Industrial Big Data Platform (IBDP). IBDP is an industrial big data platform based on a series of open source softwares for ingestion, analysis and visualization of multi-source data [9]. The model includes device templates and four strategies for data synchronization, data slicing, data splitting and data indexing, respectively. We can ingest device data from multiple sources using this heterogeneous device data ingestion model, which is verified on our IBDP. In addition, we report on a case study for a device data-based scenario analysis on the IBDP. The main contributions of our paper are the following:

1. We propose a heterogeneous device data ingestion model, which facilitates the ingestion and fusion of heterogeneous data from multiple sources.
2. We provide four data processing strategies for data synchronization, data slicing, data splitting and data indexing, respectively.
3. We implement the model on our IBDP and present a case study of data analysis after data ingestion.

The rest of the paper is structured as follows: first, a brief overview of related work is given in Section 2. The heterogeneous device data ingestion model is proposed in Section 3. Thereafter, heterogeneous sensor data ingestion methods for multiple data sources are explored in Section 4. In Section 5, the entire platform and some case studies are discussed, and finally, our conclusions are given in Section 6.

## 2. Related Work

In recent years, we’ve been witnessing explosive growth in the variety, speed, and volume of data [4]. The most fundamental challenge for the big data platform is how to explore the large volumes and analyze the data to get the useful information or knowledge for future actions [10]. The traditional data platforms are not sufficient in dealing with these challenges. Hadoop is now widely used due to its cost effective, high scalability and fault-tolerance, and now it has become the basic technology for big data [11]. Currently, many big data platform-based HDFS have been developed such as CDH, HDP, Hive, Cascade, *etc.* In our platform, HDFS is also used to store data. Liu *et al.* have summarized some of the big data platforms which have been widely used for achieving real-time availability [11]. These platforms are Hadoop Online, Storm, Flume, Spark and Spark Streaming, Kafka, Scribe, S4, HStreaming, Impala, *etc.* They are leveraged in one or more situations. They are suitable for different situations so the best effect can be achieved by integrating them.

Much research on sensor data and smart data ingestion and fusion has already been reported. Llinas *et al.* provided a tutorial on data ingestion and a basis for sensor ingestion and fusion for further study and research [12]. Regarding sensor data ingestion, various researchers have proposed frameworks; for example, Lee *et al.* presented a peer-to-peer collaboration framework for multi-sensor data fusion in resource-rich radar networks [13]. In most of these frameworks, data can be exchanged among different sensors. This is different from simple sensors, where data cannot be exchanged among the different devices. Dolui *et al.* discussed two types of sensor data processing architectures, namely, on-device and on-server data processing architectures [14]. Smart devices and products in the industrial field employ the second architecture.

For unstructured data, Sawant *et al.* summarized the common data ingestion and streaming patterns, namely, the multi-source extractor pattern, protocol converter pattern, multi-destination pattern, just-in-time transformation pattern, and real-time streaming pattern [15]. At LinkedIn, Lin Qiao *et al.* proposed the far more general and extensible Gobblin, which enables an organization to use a single framework for different types of data ingestion [16]. The structure of the data they collected is unknown, but for smart devices, the structure can be obtained if we have templates for the devices.

There are also a few specialized open-source tools for data ingestion, such as Apache Flume, Aegisthus, Morphlines, and so on, but these are generally used to ingest a single type of data. For heterogeneous device data from multiple sources, we need to ingest different types of data. Thus, we created the IBDP with a heterogeneous device data ingestion model for data from multiple sources. Using this model, we can ingest various device data and store them in a unified format.

This paper is a substantial extension of [9] in some important aspects. First, we propose a heterogeneous device data ingestion model, which facilitates the ingestion and fusing of heterogeneous data from multiple sources. Second, we provide four data processing strategies for data synchronization, data slicing, data splitting and data indexing, respectively. Third, we re-implemented the ingestion layer of IBDP which proposed in [9] with the heterogeneous device data ingestion model and the data processing strategies. Last, we provide more case study details.

## 3. Heterogeneous Device Data Ingestion Model

**Figure 2:**
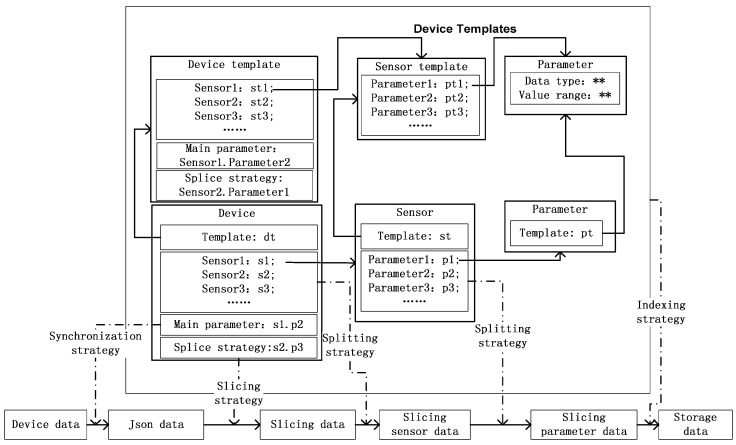
Heterogeneous device data ingestion model.

Device data include not only streaming data, but also data stored in relational databases and files. We propose a heterogeneous device data ingestion model as outlined in Figure 2. The model can receive or extract heterogeneous device from multiple sources and save them in a unified format. Included in our heterogeneous device data ingestion model are device templates and four strategies based on the device templates. The strategies cover data synchronization, data slicing, data splitting and data indexing.

### 3.1. Device Templates

Each device has sensors and each sensor has parameters. Since for a single type of device the sensors and parameters are the same, we can manage each device with templates. As shown in Figure 2, there are several sensor templates in each device template and there are several parameter templates in each sensor template. For each device, sensor, or parameter template, there may be several corresponding devices, sensors, or parameters, respectively. A device in different templates may contain the same sensor, while a sensor in different sensor templates may contain the same template parameter. In device templates, we need to set the main parameter, which is used for data synchronization. A splice strategy is also needed. Since devices may be logical, we can combine some related sensors to create a virtual device, which is also supported by the device templates.

### 3.2. Data Synchronization Strategy

Different sensor data in a device may be asynchronously transferred, especially in a virtual device. Besides, data stored in a relational database or files need to be merged, if they have been split on the basis of parameter or sensor. Thus, device data need to be synchronized. We propose a data synchronization strategy based on the main parameter in a device template. The synchronization strategy is given in Algorithm 1.

**Algorithm 1** Data Synchronization Strategy

1.	Receive data.
2.	**If** the data are asynchronously transferred
3.	   **If** the data contain the main parameter
4.	      Create a new JSONObject.
5.	      Store the data in the JSONObject according to device template.
6.	   **Else**
7.	      Store the data in the JSONObject, whose time is the closest to the time of the data.
8.	   **End If**.
9.	**Else**
10.	    Transform data into JSONObject according to device template.
11.	**End If**
12.	Output JSONObjects once a certain number of these objects have been created.



The main functions of the data synchronization strategy are data synchronization and data format conversion. To use the data synchronization strategy, we need to set the main parameter when creating the device template. When a device is added to the platform, the main parameter is automatically generated according to the templates.

For data format conversion, we need to set the data mapping relationship from the data received to the device format. For example, the format of data stored in relational databases is {*deviceId*, *sensorId*, *time*, *p1*, *p2*, *p3*}. In the process of data ingestion, each parameter value in the data item is placed in the corresponding position of the JSONObject. The format of a JSONObject is shown in Table 1.

### 3.3. Data Slicing Strategy

Since the device data are continuously updated, they should not be placed in a file. We propose a data slicing strategy for the device templates. There are three slicing methods in this strategy, the first of which is timing slicing. This method requires setting a string in the device templates, the format of which and the included parameters are given in Table 2.

For example, the string *0 4 −1 −1 −1* means that the data need to be sliced at 4:00 am every day. The second method is periodic slicing, which requires setting an integer in the device templates, denoting the length in seconds of the slicing interval. The third method involves slicing according to a specific parameter, which needs to be set in the device templates. When the parameter changes, the data are sliced. For instance, some devices need to slice data based on the value of a switch sensor. The data slicing strategy is set in the device template. When a device is added to the IBDP, it is automatically equipped with a slicing strategy. The algorithm for the data slicing strategy is given below (Algorithm 2).

**Algorithm 2** Data Slicing Strategy

1.  Obtain the data slicing strategy by deviceId in JSONObject.
2.  **If** slicing strategy is timing slicing,
       create a timer.
       When the timer reaches the specified time, create a new file and transmit the old file;
       Add data to the new file when the timer is sleeping.
    **End If**.
3.  **If** slicing strategy is periodic slicing,
       create a timer.
       At periodic intervals according to the timer, create a new file and transmit the old file;
       add data to the new file when the timer is sleeping.
     **End If**.
4.   **If** slicing strategy is slicing according to specific parameter,
       When the value of the specific parameter changes, create a new file and transmit the old file;
       add data to the new file.
     **End If**.



### 3.4. Data Splitting Strategy

In the slicing device sensor file, each data item is a JSONObject. Since the device data are heterogeneous, it is difficult to get specific parameters to analyze despite the fact that the parameters can only contain integer, float, Boolean or binary data. The device data could be used and analyzed more easily if they were split into parameters. We propose a splitting strategy based on the templates. Splitting involves two steps, the first of which is to split the file into sensor data items. Each data item in the sensor data files is listed in Table 3. The second step is to split the sensor data files into parameter data files. Each data item in the parameter data file is listed in Table 4.

### 3.5. Data Indexing Strategy

**Figure 3:**
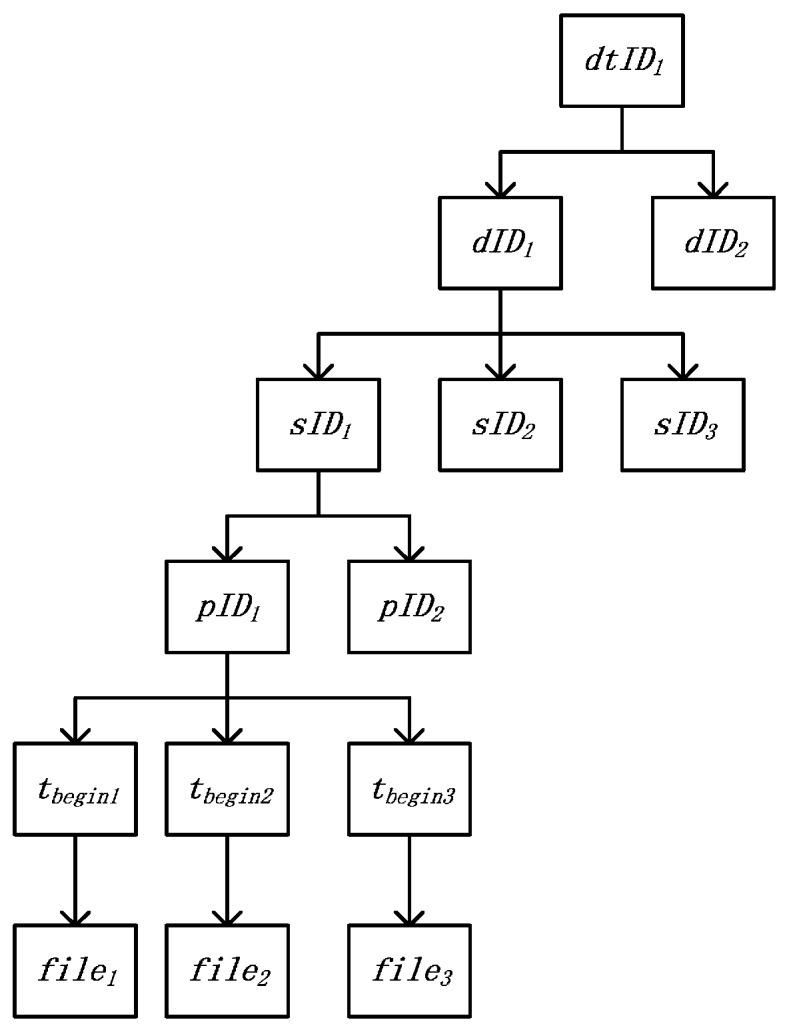
Hierarchical index of device data: *dtID* is the identity of device template, *dID* is the unique identity of device, *sID* is the unique identity of sensor, *pID* is the unique identity of parameter, and *t_begin_* is the start time in the file.

To speed up data ingestion, we propose a data indexing strategy for data saved in the Hadoop Distributed File System (hereafter, HDFS). In each file, the format of a data item is <*t*, *v*>, where *t* is the time of data generation and *v* is the value of the datum. When the files are created, they are indexed using the data indexing strategy described in Figure 3, where *dtID* is the unique identity of the device template, *dID* is the unique identity of the device, *sID* is the unique identity of the sensor, *pID* is the unique identity of the parameter, and *t_begin_* is the start time in the file. *dtID*, *dID*, *sID*, *pID*, and *t_begin_* can be obtained from the parameter data files. To save space, we use the directories of HDFS to implement the index structure. Now, we give an example to show how to use the index. Suppose that we want to get the data of one parameter whose identity is *pID_1_* from *t_begin_* (*t_begin_* is earlier than *t_begin2_* and is later than *t_begin1_*) to *t_end_* (*_end_* is earlier than *t_begin3_* and is later than *t_begin2_*) which is shown in Figure 3. First, we search the identities of the sensor and device which contain the parameter *pID_1_*. The identities are *sID_1_* and *dID_1_*, respectively. Second, we get the device template *dtID_1_* corresponding to device *dID_1_*. Third, we use directory */the_root_directory_to_store_device_data/stID_1_/dID_1_/sID_1_/pID_1_* to get data of parameter *pID_1_*. Next, we get the files *file_1_* and *file_2_* (Note that the files not only contain data items between *t_begin_* and *t_end_*, but also contain some data items earlier than *t_begin_* or later than *t_end_*). Finally, we read data items from the files, align the data items < *t*,*v* > by *t* and eliminate the data item which *t* is earlier than *t_begin_* or later than *t_end_*. After getting the data items, we can analyze the data of Section 4.4.

## 4. Heterogeneous Sensor Data Ingestion Methods

**Figure 4:**
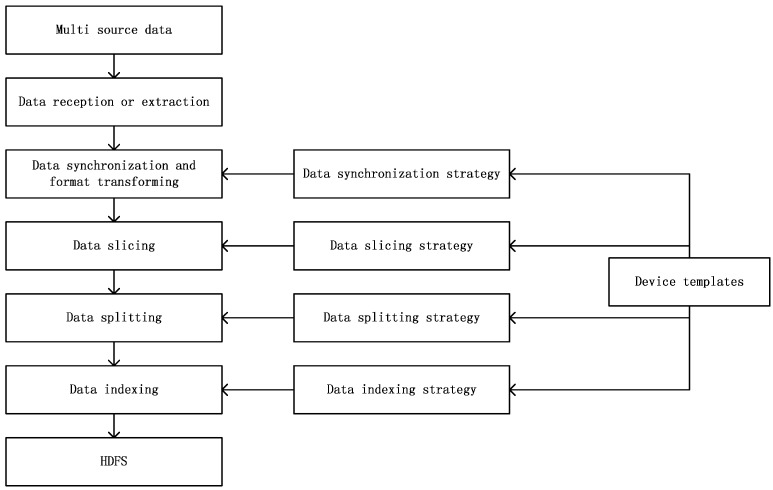
Five processes used to ingest device data.

The heterogeneous device data ingestion model includes five processes to ingest device data as shown in Figure 4. These processes are data reception or extraction, data synchronization and format transformation, data slicing, data splitting, and data indexing. The data synchronization and format transformation process corresponds to the data synchronization strategy, the data slicing process to the data slicing strategy, the data splitting process to the data splitting strategy, and the data indexing process to the data indexing strategy. The four processes for different types of data sources are the same. The data reception or extraction process differs for different types of data sources.

### 4.1. Ingestion of Device Streaming Data

Most device data comprise streaming data, which is the basic form to ingest. We do not need to extract streaming data; instead, they are received through Flume, which is a distributed, reliable, and available service for efficiently collecting, aggregating, and moving large amounts of log data. The ingestion algorithm for streaming data is given in Alogrithm 3.

**Algorithm 3** Streaming data ingestion algorithm
Receive data from the monitoring port of Flume.Receive data and convert data to a JSONObject using data synchronization strategy (described in Section 3.2, Algorithm 1).Slice the data using data slicing strategy (described in Section 3.3, Algorithm 2).Split device data files into parameter data files using data splitting strategy (described in Section 3.3).Index and store files using data indexing strategy (described in Section 3.4).


### 4.2. Ingestion Files of Device Data

Some device data are saved in the form of files after real-time analysis or original application processing. If so, we first obtain the files through Flume and process these into pseudo streaming data. Thereafter, we can view and handle the data as streaming data. The ingestion algorithm for files is given in Algorithm 4.

**Algorithm 4** Files data ingestion algorithm1.  Receive files from monitoring directories of Flume.2.  Read the data and send the data to the monitoring port of Flume.3.  Process the pseudo streaming data using streaming data ingestion algorithm (described in Section 4.1, Alogrithm 3).

### 4.3. Ingestion of Device Data from Relational Database

Some device data are stored in relational databases after real-time analysis or original application processing. To ingest the data, we first need to extract them from the relational database using JDBC and Crontab.

**Figure 5:**
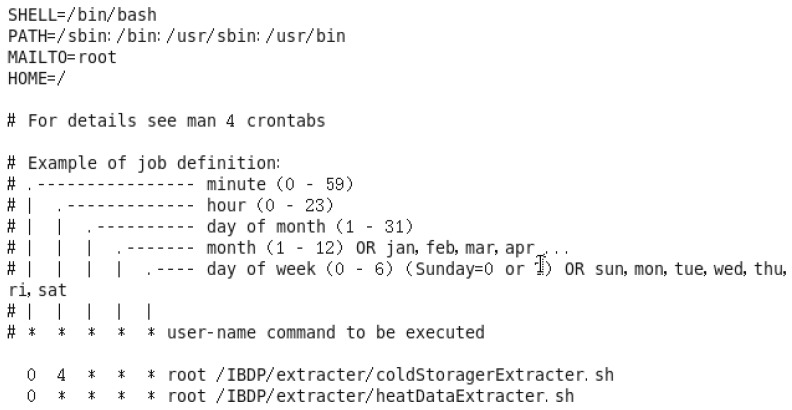
Configuration file for Crontab.

The extractor reads data from a relational database through JDBC and sends every line of data to the monitoring port of Flume. When to start the extraction is controlled by Crontab. Crontab, the configuration file for which is shown in Figure 5, is a command provided by the operating system. When we create a timing job, a new line is added to the end of the file. Each line in Crontab, the description of which is shown towards the middle of Figure 5, is a command to start an extractor. Two command examples are given at the bottom of Figure 5. The first command, 0 4 * * * root /IBDP/extracter/coldStoragerExtracter.sh means that the system will start an extractor as described in file *coldStoragerExtracter.sh* at 4:00 am.

The data after ingestion by the extractor take the form of pseudo streaming data, which can be viewed and handled as streaming data. The ingestion algorithm for relational data is given in Algorithm 5.

**Algorithm 5** Relational data ingestion algorithm
Start up an extractor using Crontab.Read the data in relational database through JDBC and send the data to the monitoring port of Flume.Process the pseudo streaming data using streaming data ingestion algorithm (described in Section 4.1, Alogrithm 3).


### 4.4. Analysis of Device Data

**Figure 6:**
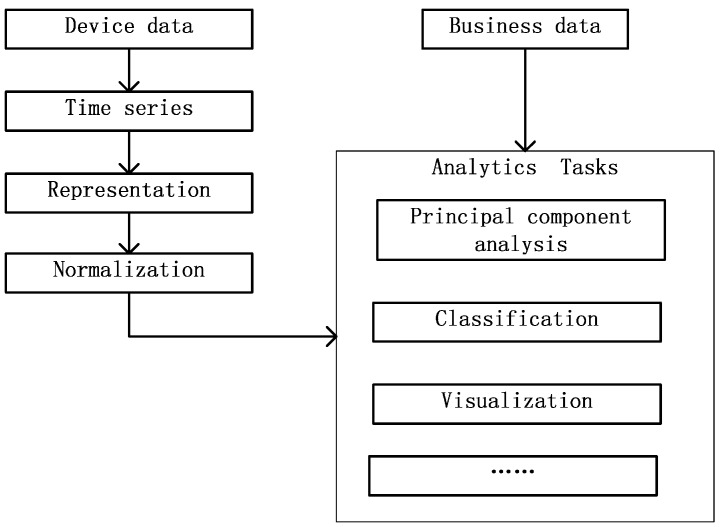
Data analysis process.

The device data are stored in a unified format after ingestion. However, in most cases, at least one item of the device data is meaningless. Recent empirical evidence strongly suggests that we should analyze the data as a time series. Moreover, the device data need to be fused with business data. In most cases, we analyze the data according to the process outlined in Figure 6.

Though the device data are in a unified format after ingestion, there are still some difficulties in analyzing them, for example, the data frequency may not be consistent. Time series with different time intervals should not be compared directly. To support data for enterprise analysis, we provide a library of algorithms on IBDP, containing various common algorithms for time series including:

(1) Representation algorithms. In IBDP, there are several time series representation algorithms, such as sampling, piecewise aggregate approximation [17], discrete Fourier transforms [18], discrete wavelet transforms [19], piecewise linear representation [20] and piecewise linear representation based on series importance point [21]. After choosing a representation, the time series can be compared with each other.
(2) Normalization algorithms. The time series obtained are generally accompanied by much noise. The IBDP contains the min-max and Z-score normalization algorithms. After normalization, the impact of noise is greatly reduced.
(3) Principal component analysis algorithms. We do not need to analyze all the parameters of each type of device; instead, only key parameters are extracted for analysis. The IBDP includes a principal component analysis [22] algorithm to analyze the principal components of a time series.
(4) Visualization algorithms. To better display the features to enterprises, there are line chart [23] and ThemeRiver [24] algorithms for time series visualization. Using these visualization algorithms, enterprises can see the features directly.
(5) Classification algorithms. The IBDP includes some classification (as well as early classification) algorithms. The main classification algorithms are shapelets (perfectly accurate shapelets from [25], and fast shapelets from [26]), 1NN, and early classification of time series [27]. Using the classification algorithms, enterprises can classify time series for different analytical tasks.

In the analytical tasks, we use not only device data, but also business data. By fusing the data, the analytical result is more accurate and more useful.

## 5. Case Study of Industrial Data Analysis

### 5.1. IBDP: An Industrial Big Data Platform

**Figure 7:**
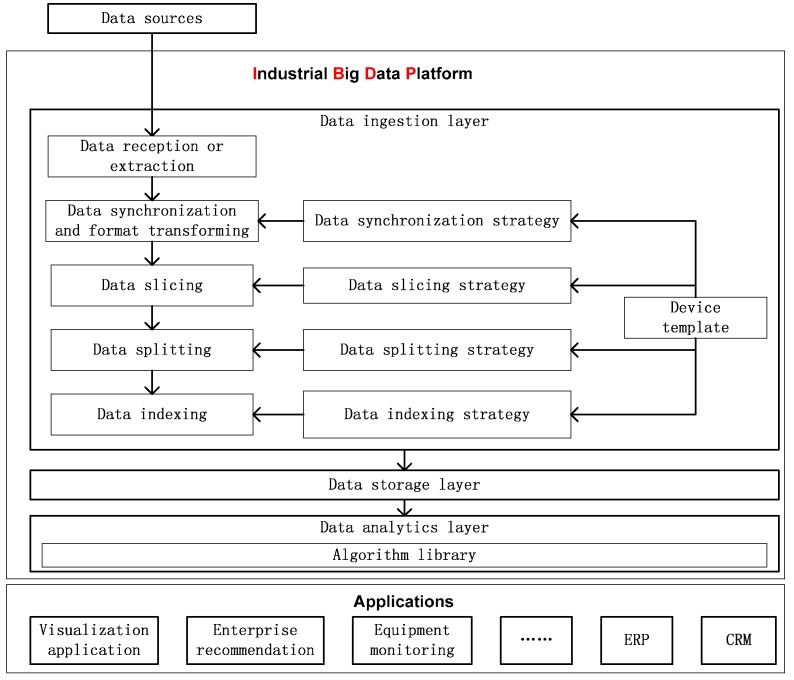
Architecture of IBDP.

The high-level architecture of a big data platform consists of three main layers: the data ingestion layer, data analytic layer, and data storage layer [4]. We implemented the heterogeneous device data ingestion model and methods in the IBDP [9], the architecture of which is shown in Figure 7.

Data are saved in the data storage layer and analyzed in the data analytic layer. There are a series of applications based on the IBDP and supported by analysis results.

The platform and applications are deployed on the cloud resource center, which allows us to adjust the storage capacity and computation ability at any time according to the need [28,29,30]. The extractors, Flume agents, and other processes for data ingestion can be extended, as can the storage capacity of the HDFS, and the computing ability of Spark and MapReduce.

We used our platform to ingest data from the Longda Foodstuff Group Co., Ltd. (Jinan, China, hereafter refrerred to as Longda,) [31] and the Jinan District Heating Limited Company (Jinan, China hereafter JDH,) [32]. In Longda, there are nearly 200 production devices, from which we obtained the operating data. We also created 22 virtual devices for cold storage. JDH monitors the heating in 14,237 apartments and we created one virtual device for all the apartments. All the virtual heating monitoring devices point to the same template. The devices are summarized in Table 5.

In the near future, we will also ingest production device data from Zhongtong Bus Holding Co., Ltd. (Jinan, China, hereafter, ZTB) [33] as well as the operational data of buses they manufacture. We will also ingest heating data from Jinan Theral Power Co., Ltd. (Jinan, China, hereafter JTP) [34]. In addition, we would like to use the platform for other companies’ data.

### 5.2. Case Study Analyzing Temperature Sensor Data

**Figure 8:**
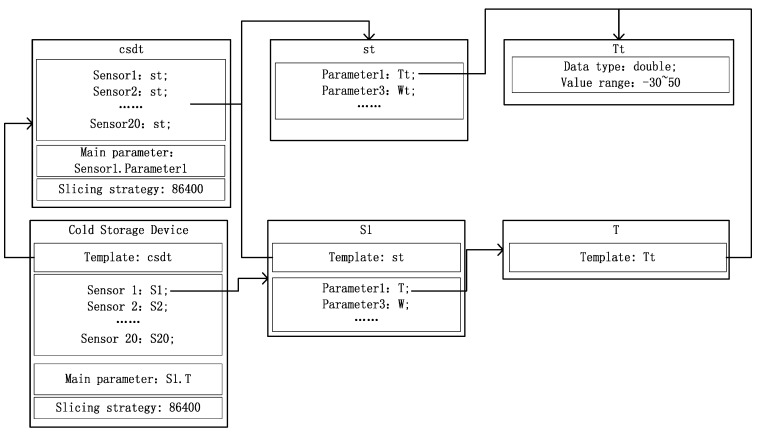
Virtual cold storage device.

In Longda, products are kept in several cold storage rooms, each of which has 20 refrigeration units to maintain the temperature. Each refrigeration unit has a sensor, which is used to monitor the temperature in the cold storage (T) room and electric power consumption (W). We use these sensors in the cold storage room to construct a virtual smart device as shown in Figure 8. Note that only Sensor 1 (S1) is shown as sensors S1 to S20 are all the same.

In each cold storage room, the main parameter is T in Sensor 1 and the slicing strategy is 86,400, which means we slice data every 86,400 s (once a day).

**Figure 9:**
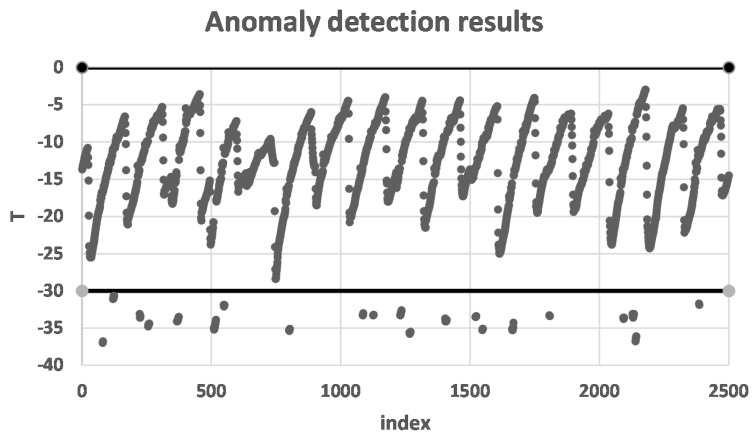
Anomaly detection results.

After the data had been stored, 2500 data items of T were obtained by indexing. We used a threshold detection algorithm for detection because this would affect the quality of the products when the temperature was too high or too low. The high threshold is 0 and the low threshold is −30 as the two horizontal lines in Figure 9. The anomaly detection results are shown in Figure 9, where the points below the horizontal line of −30 are abnormal owing to too low temperatures.

**Figure 10:**
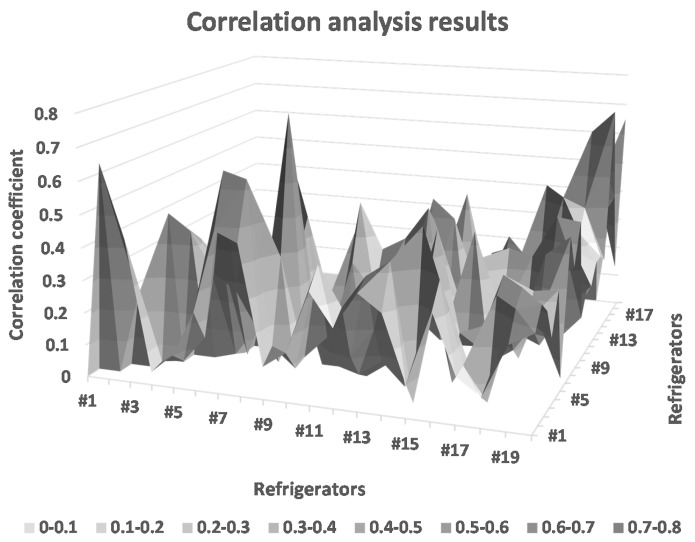
Correlation analysis results of some cold storage rooms.

Thus, we obtained T parameters, which were affected by the temperature of the cold storage and the working status of the refrigeration units. Then, we analyzed the correlation between them using a near correlation coefficient. The correlation analysis results are shown in Figure 10.

From Figure 10, we can see the working status of the refrigeration unit affecting the value of T the most. The working status of refrigerators #1 and #2, #3 and #4, …, #19 and #20 is similar.

The price of electricity differs at different times. The price at night is much lower than that during the day. The prices are shown in Table 6. To reduce cost, we expect that cooling happens between 23:00 and 07:00.

We analyzed the relation between electric power consumption and temperature. When the temperature is between 0 and −30, the fitting functions are as shown in Table 7.

Finally, we formulated the strategy for refrigeration, which begins at 23:00 and lasts for *3 + (t_open_)/2* h, where *t_open_* is the total open time.

## 6. Conclusions

With the development of digitized manufacturing, data ingestion and analysis have become more and more important. In this paper, we presented a data ingestion model for heterogeneous devices, which consists of device templates and four strategies for data synchronization, data slicing, data splitting, and data indexing, respectively. Next, we introduced heterogeneous sensor data ingestion methods to ingest device data from multiple sources. Finally, we presented a case study describing a scenario in which device data were analyzed on the IBDP. We showed that the heterogeneous device data ingestion model can be used to ingest, analyze, and store data. It also makes it easier and more efficient for enterprises to analyze data.

## Figures and Tables

**Table 1:** Format of JSONObject.

{ “deviceId”: “A000000100000001”; “deviceTemplateId”: “A0000001”; “time”: 2016-01-31 18:30:06.0; “sensorData”:[ { “sensorId”: “B000000000000001”; “parameterData”:[ { “parameterId”: “C000000000000001”; “value”: 10.3}, { “parameterId”: “C000000000000002”; “value”: 21.4}, .... ] }, { “sensorId”: “B000000000000002”; “parameterData”:[ { “parameterId”: “C000000000000003”; “value”: 14.2}, { “parameterId”: “C000000000000004”; “value”: 12.2}, .... ] }, … ] }

**Table 2:** Format of string and included parameters for timing slicing.

Parameter	Description
String format	String containing five integer parameters, *m h d M dw*, in order, for example, 0 4 −1 −1 −1.
*m*	Minute, ranging from −1 to 59 (−1 means that minute has not been set)
*h*	Hour, ranging from −1 to 23 (−1 means that hour has not been set)
*d*	Day of month, ranging from −1,1 to 31 (−1 means that day of month has not been set)
*M*	Month, ranging from −1,1 to 12 (−1 means that month has not been set)
*dw*	Day of week, ranging from −1 to 6 (−1 means that day of week has not been set; Sunday = 0, Monday = 1, ..., Saturday = 6)

**Table 3:** Format of data items in sensor data files.

{ “deviceId”: “A000000100000001”; “deviceTemplateId”: “A0000001”; “time”: 2016-01-31 18:30:06.0; “sensorId”: “B000000000000001”; “parameterData”: [ { “parameterId”: “C000000000000001”; “value”: 10.3}, { “parameterId”: “C000000000000002”; “value”: 21.4}, .... ] }

**Table 4:** Format of data items in parameter data files.

{ “deviceId”: “A000000100000001”;“deviceTemplateId”: “A0000001”; “time”: 2016-01-31 18:30:06.0;“sensorId”: “B000000000000001”; “parameterId”: “C000000000000001”; “value”: 10.3 }

**Table 5:** Devices currently ingested by IBDP.

Device	Number	Type of Data	Company
production devices	almost 200	streaming data	Longda
virtual cold storage devices	22	file	Longda
virtual heating monitoring devices	14,237	relational database	JDH

**Table 6:** Price of electricity at different times.

Time	Price
10:30–11:30, 19:00–21:00	1.2773
08:30–10:30, 18:00–19:00, 21:00–23:00	1.2068
07:00–08:30, 11:30–18:00	0.7838
23:00–07:00	0.3608

**Table 7:** Fitting functions when the door of the cold storage is closed.

Status	Temperature (T) and Time (t)	Power (W) and Time (t)
No refrigeration and door of cold storage is closed	T‘ = T + 1.5 × t	W‘ = W + 11 × t
No refrigeration and door of cold storage is open	T‘ = T + 5.4 × t	W‘ = W + 11 × t
Refrigeration and door of cold storage is closed	T‘ = T − 10.95 × t	W‘ = W+3225 × t

## Abbreviations

The following abbreviations are used in this manuscript:

IBDP: Industrial Big Data Platform
IoT: Internet of Things
Longda: Longda Foodstuff Group Co., Ltd.
JDH: Jinan District Heating Co., Ltd.
ZTB: Zhongtong Bus Holding Co., Ltd.
JTP: Jinan Theral Power Co., Ltd.
